# The relationship between occupational stigma consciousness and occupational identity among pre-service early childhood education teachers in China: a four-year longitudinal study

**DOI:** 10.3389/fpsyg.2026.1793509

**Published:** 2026-03-17

**Authors:** Xiaoling Ren, Ru Feng, Zhonglian Yan, Jiaxin Liu, Ziyi Guo

**Affiliations:** 1 School of Education, Beihua University, Jilin, China; 2 Faculty of Education, Northeast Normal University, Changchun, China

**Keywords:** longitudinal study, occupational identity, occupational stigma consciousness, pre-service early childhood education teachers, teacher education

## Abstract

**Introduction:**

For pre-service early childhood education teachers, occupational identity is foundational to career commitment. However, its cultivation is challenged by occupational stigma consciousness. Longitudinal research on their reciprocal relationship remains limited. This study examined this gap over 4 years.

**Methods:**

A four-wave longitudinal survey involved 34 pre-service early education teachers from University B in Jilin Province, China. Participants completed the Occupational Stigma Scale and Occupational Identity Scale. Repeated measures ANOVA examined time, gender, and admission status effects. Cross-lagged panel analysis tested reciprocal relationships between occupational stigma consciousness and occupational identity.

**Results:**

(1) both variables were unstable; occupational stigma consciousness peaked in the freshman year, lowest in junior year; occupational identity was lowest in freshman year, significantly lower in sophomore than junior year; (2) males and reassigned admission students reported higher stigma and lower identity; (3) earlier occupational stigma consciousness negatively predicted later occupational identity; conversely, freshman occupational identity negatively predicted sophomore stigma, but subsequent years showed no prediction.

**Discussion:**

Findings underscore the urgent need to mitigate occupational stigma consciousness impacts on pre-service early education teachers' occupational identity, especially during initial training stages where identity's protective role is most evident against stigma.

## 1 Introduction

High-quality early childhood education fundamentally requires well-qualified teachers, yet pre-service teacher training in early childhood education urgently needs improvement. While research shows that strong occupational identity predicts their learning engagement, occupational confidence, and teaching motivation (Hong et al., 2017; Forsythe, 2005), persistent societal biases undermine this occupation. The prevalent tendency to label early childhood teachers as “childminders,” “babysitters,” or “play supervisors” (Qing, 2009), and to ask, “Don't they just watch and play with children? Do they even need lesson plans? Does this job require real occupational skills?” (Eidin and Shwartz, 2023; Zhang and Xu, 2021), not only diminishes their status but also devalues their occupational preparation, placing the field at the bottom of the educational hierarchy.

In recent years, highly publicized child abuse cases have triggered significant public outrage. Perpetrators are frequently labeled as “morally degraded” or “monsters” (Bo et al., 2022; Moreno, 2022). This condemnation has extended to the early childhood education occupation itself, casting doubt on teachers' overall professionalism and ethics (Zhang L. et al., 2020) and substantially harming their public image (Moreno, 2022). Through repeated negative coverage, the media shapes public perception, thereby contributing to occupational stigmatization.

As part of society, higher education institutions are not immune to these influences. It is therefore crucial to examine the relationship between pre-service early childhood teachers' occupational identity and their consciousness of occupational stigma. Previous research, predominantly cross-sectional, has not adequately explored the longitudinal or bidirectional nature of this relationship. A key question of directionality remains: Does heightened stigma consciousness erode occupational identity over time, does a weak identity increase sensitivity to stigma, or do they interact in a reciprocal cycle?

This study addresses this gap within the Chinese context. Occupational stigma in early childhood education is a concern in many non-Western societies. Understanding the Chinese case can thus contribute to a broader, cross-cultural comprehension of teacher development under conditions of occupational devaluation. To clarify this dynamic, we employ a cross-lagged panel design. This longitudinal method measures both variables at multiple time points. It allows for the assessment of bidirectional relationships while statistically controlling for prior levels of each variable (i.e., autoregressive effects) and their baseline association (Chen et al., 2025). This approach is suited to untangling the direction of influence between occupational stigma consciousness and occupational identity over time.

### 1.1 The emergence and evolution of occupational stigma

The phenomenon of occupational stigma has existed for a long time (Shantz and Booth, 2014). Occupational stigma comes from the public's negative stereotypes about certain occupations. These stereotypes relate to job representation, ethical standards, and social relationships (Ashforth et al., 2007). In terms of job representation, some occupations face stigma because they involve frequent exposure to pollutants or hazardous materials, such as butchers and morticians (Meara, 1974). In terms of ethical standards, stigma arises when an occupation routinely involves content that violates widely accepted moral norms, such as strippers and ticket scalpers (Kamise, 2013). In terms of social relationships, stigma occurs when an occupation requires individuals to compromise their dignity to serve others or frequently engage with marginalized groups, such as servers and prison guards (Benjamin et al., 2011). These stereotypes not only show aspects of the occupations but also reveal the public's cognitive biases. When an occupation is stigmatized, practitioners are often aware of the threat posed by negative stereotypes. This forms varying degrees of occupational stigma consciousness (Shantz and Booth, 2014). A person's experience of occupational stigma consciousness depends on how recognizable the stigma is and the individual's capacity for self-regulation (Crocker et al., 1991).

Research on occupational stigma has primarily focused on traditional low-prestige occupations perceived as “dirty work,” such as sanitation workers, sex workers, and domestic helpers (Pereira et al., 2021). These occupations frequently engage with “stigmatized” individuals, matters, or objects. As a result, they often face public disdain, aversion, and avoidance (Devers et al., 2009). When workers in low-prestige occupations perceive the threat of occupational stigma, they may develop negative behaviors and psychological states. These include turnover intentions, emotional exhaustion, and high-stress levels (Hughes, 1958). However, in reality, almost all occupations involve some degree of “stigma” and consequently suffer varying levels of stigmatization (Kreiner et al., 2006). This recognition has gradually expanded the scope of occupational stigma research. It now includes high-prestige occupations, such as physicians, lawyers, and teachers (Zhou and Huang, 2018). Nevertheless, the impact of occupational stigma consciousness on individuals in high-prestige occupations remains inconclusive. Some studies suggest that practitioners' social status correlates positively with their occupational prestige (Press, 2021). The decline in social identity triggered by occupational stigma among high-prestige occupational groups may be partially offset by their elevated occupational prestige. This compensation could weaken the negative effects of occupational stigma consciousness (Stenross and Kleinman, 1989). Conversely, other research indicates that for high-prestige occupationals, the sense of shame induced by occupational stigma often creates a stark contrast with the honor derived from their high-status positions (Kreiner et al., 2006; McMurray and Ward, 2014). This contrast inevitably leads to significant psychological dissonance. It can substantially negatively impact their occupational psychology, affect, and behaviors (McMurray and Ward, 2014).

### 1.2 Occupational stigma consciousness of early childhood education teachers

In China, the teaching occupation has consistently been regarded as one of the most prestigious occupations (Li et al., 2022). However, the image of early childhood education teachers is currently facing challenges. These include discrepancies in public perception, a decline in real-world reputation, and shifts in media portrayal (Wu and Zhang, 2017). This situation has led to descriptions of early childhood education teachers characterized by “two lows and one poor”: low occupational ethics, low technical skill requirements in job content, and poor personal qualities (Zhang L. et al., 2020). These perspectives reflect a negative public image. This suggests that the occupation has experienced a degree of stigmatization. Empirical assessments have revealed that occupational stigma consciousness among early childhood education teachers in China is above moderate (Bo et al., 2022).

Furthermore, the origins of occupational stigma enable analysis of potential stigmas linked to early childhood teaching. These can be viewed through job representation, ethical standards, and social relationships. Firstly, early childhood education teachers are responsible for both education and childcare. They often assist children with dressing and feeding, which are usually considered low-skilled service tasks. This may cause stigmatization in social relationships (Gu et al., 2020). Secondly, these teachers encounter children's excreta and vomit at work. Their environment is noisy and involves contact with unclean materials, leading to stigmatization regarding job representation (Bo et al., 2022). Thirdly, child abuse is despised and unforgivable to the public. Incidents involving early childhood education teachers can trigger suspicion toward all teachers, risking ethical stigma (Vough et al., 2013). To our knowledge, no empirical study has assessed occupational stigma consciousness in pre-service early childhood education teachers.

### 1.3 Occupational identity of pre-service early childhood education teachers

The connotation of occupational identity includes psychological components like affect, cognition, volition, and behavior. It is closely linked to occupational motivation, values, career commitment, and orientation (Rodrigues and Mogarro, 2019). Increasingly, occupational identity is recognized not only as an outcome but as a critical psychological resource that sustains teachers. It is a key predictor of work engagement and overall well-being (Yu et al., 2025). Based on these, pre-service teachers' occupational identity refers to their perceptions and experiences as students and future teachers (Beauchamp and Thomas, 2011; Moussay et al., 2011). Measurement relies mainly on individually developed scales, such as the “Pre-service Teacher Occupational Identity Scale” (Wang et al., 2010). Surveys show room for improvement in current levels of pre-service teacher occupational identity, with significant gender and admission status differences (Huang, 2021; Wang, 2015). Admission status can be categorized into two types: reassigned and non-reassigned admission. Reassigned admission is a mandatory procedure in China's enrollment system for students who meet the institutional threshold but do not meet the cutoff for their chosen majors, placing them in other programs with open spots. Non-reassigned admission means students meet the cutoff for their selected majors.

Researchers primarily categorize the influencing factors of pre-service teacher occupational identity into individual and environmental factors (Johnson and Ridley, 2018). Individual factors mainly include personal knowledge, perspectives, experiences, and aspirations, such as learning engagement, teaching motivation, and career preparation (Thomas and Beauchamp, 2011). Environmental factors at the macro level primarily encompass policy, cultural, and societal elements, such as educational policies and social status. In contrast, at the micro level, they mainly refer to family and school influences, such as family support and education quality (Thomas and Beauchamp, 2011). Furthermore, some studies have found that, among the factors influencing pre-service teachers' occupational identity, societal factors are significantly more influential than school and family factors (Beijaard et al., 2004; Thomas and Beauchamp, 2011).

### 1.4 Relationship between occupational stigma consciousness and occupational identity

Research on the relationship between occupational stigma consciousness and occupational identity has primarily analyzed the impact of the former on the latter. Both occupational stigma consciousness and occupational identity coexist within an individual's perception of their occupation (Kamise, 2013). Faced with these two contradictory perceptions and experiences, practitioners may find themselves in a dilemma, caught between a desire for recognition and the difficulty of obtaining it (Gurr, 1970). Ashforth and Kreiner (1999) proposed a theoretical framework suggesting that occupational stigma consciousness can generate ambivalent psychological reactions toward one's occupation among its practitioners (Ashforth and Kreiner, 1999). Subsequently, some studies have indicated that occupational stigma is undoubtedly a pain point for practitioners, easily generating feelings of occupational stress and inferiority, and even leading to emotional exhaustion and diminished core self-evaluation, thereby hindering the construction of a positive occupational identity (Kreiner et al., 2006; Miller and Kaiser, 2001). However, other studies have failed to identify a significant correlation between occupational stigma consciousness and occupational identity (Shantz and Booth, 2014), with some studies even finding that occupational stigma consciousness can, conversely, stimulate practitioners' occupational self-esteem and promote proactive coping strategies to defend their occupational identity, such as positive resistance and selective social status comparison (Ashforth and Kreiner, 1999; Hughes et al., 2017).

### 1.5 Theoretical framework

This study utilizes three theoretical frameworks to explain the longitudinal interaction between occupational stigma consciousness and occupational identity. These theories illustrate how pre-service early childhood education teachers perceive and respond to the societal devaluation of their occupation.

First, Lazarus and Folkman's transactional model explains stress and coping processes (Folkman and Lazarus's, 1985). In this model, occupational stigma consciousness triggers a primary appraisal, where individuals evaluate whether societal stigma threatens their occupational self-concept. This is followed by a secondary appraisal, in which they assess coping resources and select strategies. Coping strategies range from problem-focused approaches, such as skill enhancement, to emotion-focused approaches, like cognitive distancing. These coping mechanisms subsequently influence the development of occupational identity, explaining how stigma consciousness may negatively predict occupational identity over time.

Second, Savickas's career construction theory provides a developmental perspective by framing occupational development as an ongoing narrative process (Savickas, 2019). Individuals construct coherent occupational stories from life experiences. For pre-service early childhood education teachers, stigma consciousness presents a challenge to occupational adaptability, necessitating narrative processing, and meaning-making. Adaptive strategies may include reframing stigma as evidence of the occupation's importance, emphasizing personal values in contrast to societal perceptions, or, in some cases, leading to narrative foreclosure through occupational abandonment. Early narrative reconstruction may buffer perceptions of stigma, suggesting that a stronger occupational identity could predict lower stigma consciousness. However, as personal narratives stabilize, this buffering effect may diminish.

Third, Festinger's cognitive dissonance theory addresses the motivational tension between occupational commitment and societal devaluation (Festinger, 1957). When pre-service early childhood education teachers maintain a positive occupational identity while being aware of occupational stigma, they experience psychological dissonance. This aversive state motivates efforts to restore consistency, such as devaluing one's occupational identity or discounting the legitimacy of stigma (Major and O'brien, 2005). The choice of strategy may shift during training, influenced by the salience of stigma and prior commitment. Thus, dissonance theory offers a key motivational mechanism for understanding longitudinal change and directly informs hypotheses regarding why one construct may predict changes in the other.

Integrating these perspectives, this study conceptualizes a dynamic and reciprocal relationship between occupational stigma consciousness and occupational identity. Stigma consciousness acts as a chronic stressor, prompting appraisal, and coping processes that shape identity. Concurrently, it poses a narrative challenge to one's occupational story, requiring adaptive meaning-making. The psychological tension between identity and stigma also generates cognitive dissonance, motivating efforts to reduce inconsistency. This integrated framework accounts for core motivational and perceptual dynamics. To further contextualize the observed developmental trajectories, the discussion will also engage complementary perspectives, such as occupational socialization stage theory and reality shock theory. Together, this framework explains the expected longitudinal dynamics and provides theoretical grounding for examining bidirectional relationships. Testing this reciprocal and adaptive process requires a method capable of modeling bidirectional predictions over time, for which the cross-lagged panel model is well-suited.

### 1.6 Present research

Building on this framework, the current investigation utilized a four-wave longitudinal design to examine the dynamic interplay between occupational stigma consciousness and occupational identity in a sample of pre-service early childhood education teachers. The study tested four hypotheses:

H1. Both constructs would demonstrate significant temporal stability across measurement waves.

H2. gender and admission status differences would emerge in these constructs;

H3. Higher initial levels of occupational stigma consciousness would predict subsequent decreases in occupational identity.

H4. Higher initial levels of occupational identity predict subsequent reductions in occupational stigma consciousness.

By adopting a cross-lagged design, this research aims to elucidate potential bidirectional pathways linking these variables, thereby offering evidence-based insights to enhance teacher preparation programs in early childhood education.

## 2 Materials and methods

### 2.1 Participants

This study was conducted at University B, a provincial comprehensive university in Northeast China with a long-standing tradition in teacher education. The research took place within the university's School of Education Science. This School serves as a designated regional center for teacher education resources and operates as an official provincial training base for the National Teacher Training Program. Participants were undergraduate students enrolled in the School's 4-year Early Childhood Education program. Launched in 2009, this program is among the earliest university-based early childhood teacher education tracks established in China's regional higher education sector. It operates within the national first-batch teacher education admissions system, follows the standardized national curriculum, and awards graduates a Bachelor of Education degree. The program's annual enrollment is typical for comparable institutions. For example, the 2020 cohort consisted of 52 students, which reflects the common intake size for such programs in recent years. A provincial-level teaching team delivers academic instruction. A structured practicum network with partner kindergartens further supports the program. It is housed within an Education discipline that has been formally recognized as a provincial high-level characteristic discipline.

A multi-stage convenience sampling strategy was used to recruit participants from the 2020 cohort. A four-wave longitudinal survey was administered to track occupational stigma consciousness and occupational identity. In the first survey wave (Time _1_), 48 questionnaires were distributed, yielding 43 valid responses after screening, for a valid response rate of 89.58%. The second wave (Time _2_) collected 42 questionnaires, of which 40 were valid (response rate: 95.23%). The third wave (Time _3_) yielded 40 questionnaires, with 39 valid responses (response rate: 97.5%). The fourth wave (Time _4_) collected 39 questionnaires, all of which were valid (response rate: 100%).

A total of 34 participants completed all four waves and were included in the final longitudinal sample, representing an overall retention rate of 79.07% throughout the 4-year study period. The sample comprised 8 male and 26 female participants. Thirteen participants were reassigned admissions, and twenty-one were non-reassigned admissions. Regarding geographical background, 18 participants were from rural areas and 16 from urban areas. Regarding their parental education levels, 12 fathers and 9 mothers had completed technical secondary education or higher (a form of secondary vocational education in China lasting typically 3–4 years post-junior high school). In comparison, 22 fathers and 25 mothers had educational attainment below the level of technical secondary school.

## 3 Measures

### 3.1 Occupational identity scale

The study employed an adapted version of Wang et al.'s (2010) Student Occupational Identity Scale (Wang et al., 2010). Previous Chinese studies on pre-service early childhood education teachers' occupational identity have successfully employed this scale (Gao, 2019; Xie and Yin, 2022). The scale comprises 12 items. Responses were recorded on a 5-point Likert scale (1 = strongly disagree, 5 = strongly agree), including one reverse-scored item. Higher scores indicate stronger occupational identity. The scale demonstrated good reliability across all four waves (Cronbach's α = 0.82, 0.85, 0.81, 0.84).

### 3.2 Occupational stigma consciousness scale

The study utilized the Occupational Stigma Consciousness Scale, initially developed by Pinel (1999; revised 2005; Pinel, 1999; Pinel and Paulin, 2005). This scale was later translated and adapted by Zhou and Huang (2018) for use in high-prestige occupations in the Chinese context (Zhou and Huang, 2018). Bo et al. (2022) successfully applied this instrument to early childhood education teachers (Bo et al., 2022). The 6-item scale was slightly modified for early childhood education students. Responses were measured using a 5-point Likert scale, with higher scores indicating greater stigma consciousness. The scale demonstrated acceptable reliability across all waves (Cronbach's α = 0.70, 0.72, 0.71, 0.75). To examine the construct validity of the scale within our specific sample, a confirmatory factor analysis (CFA) was conducted using Time 1 data (the largest wave). The single-factor model showed marginally acceptable fit to the data: χ^2^/df = 2.15, CFI = 0.95, TLI = 0.92, RMSEA = 0.08, SRMR = 0.05. While the RMSEA was slightly above the ideal threshold, other indices suggested the unidimensional structure of the scale was largely tenable in this sample of pre-service early childhood education teachers.

### 3.3 Procedure

This study employed a 4-year longitudinal panel design to collect annual data on occupational stigma consciousness and occupational identity among pre-service early childhood education teachers through electronic questionnaires. Project coordinators facilitated participant recruitment from collaborating universities. All procedures across the four waves of data collection strictly adhered to established research ethics protocols. Before each assessment, participants were reminded of the voluntary nature of their participation, the anonymity of their responses, and their right to withdraw at any time. During each data collection session, the electronic questionnaire link was provided in the classroom along with standardized instructions. Researchers addressed participants' clarification questions and reminded them to submit their responses promptly. To minimize potential coercive pressure, researchers maintained a non-intrusive presence at the back of the classroom and deliberately avoided monitoring individual participants or observing their reactions.

To enable anonymous longitudinal tracking of participants across the four survey waves, each individual was assigned a unique code derived from the last six digits of their Resident Identity Card number in the People's Republic of China. Participants entered this code on each electronic questionnaire during every measurement wave. All assessments were deliberately scheduled outside major Chinese public holidays, with approximately 1 year between waves. The specific time points were as follows: Time _1_ (T_1_) on December 1, 2020; Time _2_ (T_2_) on December 3, 2021; Time _3_ (T_3_) on November 30, 2022; and Time _4_ (T_4_) on November 28, 2023.

Following each wave of data collection, a multi-step screening procedure was applied. First, questionnaires with more than 15% missing responses were excluded. Second, surveys with patterned or invariant responses (e.g., selecting the same option across all items) were flagged and removed. Third, questionnaires completed in less than 5 min were considered invalid and eliminated. Finally, only participants who completed all four waves and could be accurately matched via their unique codes were retained for longitudinal analysis. The study protocol was approved by the Ethics Committee of the College of Education at Beihua University (Approval No. BHSE-2025-313). It was conducted in full accordance with the ethical principles outlined in the Declaration of Helsinki.

### 3.4 Analytical strategy

The data analysis proceeded in two phases. Initially, all valid responses obtained across the four measurement waves were imported into SPSS 27.0. Descriptive statistics were computed for every variable at each wave. To evaluate the potential influences of gender, admission status, and time, separate two-way repeated-measures analyses of variance were conducted for occupational stigma consciousness and occupational identity, and time (T1, T_2_, T3, T4) served as the within-subjects factor. In contrast, gender and admission status constituted the between-subjects factors. Where the assumption of sphericity was not met, Greenhouse–Geisser corrections were employed. The conventional *p* < 0.05 threshold was used to determine statistical significance.

To test our longitudinal hypotheses regarding reciprocal prediction, we employed a traditional cross-lagged panel model. This analytical approach was appropriate for three reasons. First, it directly tests whether one construct prospectively predicts later levels of another, after controlling for prior levels of the outcome variable. This aligns with our aim to examine bidirectional predictions. Second, it is well-suited for modeling developmental change across fixed, meaningful intervals, such as the 4-year training program we use. Third, it provides a clear and interpretable baseline of temporal dynamics in an under-researched context, establishing a foundation for future research.

Before estimating this model, we examined longitudinal measurement invariance for both constructs across the four waves using Mplus 8.0. Model fit was assessed using multiple indices: χ^2^/df, Comparative Fit Index (CFI), Tucker–Lewis Index (TLI), Standardized Root Mean Square Residual (SRMR), and Root Mean Square Error of Approximation (RMSEA). Measurement invariance was considered tenable if differences in fit indices between nested models met conventional thresholds (ΔCFI ≤ 0.015, ΔRMSEA ≤ 0.01).

Thereafter, the four-wave cross-lagged panel model was specified and estimated in Mplus 8.0. The model incorporated both autoregressive (stability) paths and cross-lagged (predictive) paths. Specifically, earlier levels of occupational stigma consciousness were modeled as predictors of subsequent occupational identity, and vice versa. Results are reported using standardized regression coefficients to indicate the magnitude and direction of these longitudinal associations.

## 4 Results

### 4.1 Common method bias

To address potential common method bias arising from self-reported questionnaires completed by pre-service early childhood education teachers, a Harman's single-factor test was employed. The test was conducted across four measurement occasions. The variance explained by the first factor for these four waves was 24.89, 37.39, 21.91, and 38.98%, all of which fell below the 40% critical threshold (Baumgartner et al., 2021). Consequently, the results indicate that common method bias is not a substantial concern for the data used in this study.

### 4.2 Measurement invariance analysis

This study conducted longitudinal measurement invariance tests across four time points using the Occupational Identity Scale and Occupational Stigma Consciousness Scale. The results indicated that both scales demonstrated strong invariance model fit, with minimal changes in CFI and RMSEA between nested models, satisfying the criteria for invariance determination (see Table 1). These findings confirm the high stability of both scales during the longitudinal measurement period, thereby providing robust support for subsequent longitudinal analyses.

**Table 1:** Test for longitudinal measurement invariance.

**Variable**	**Model**	**χ^2^/df**	**CFI**	**TLI**	**RMSEA**	**RMR**	**Model comparison**	**ΔCFI**	**ΔRMSEA**
OI	M1	2.01	0.99	0.99	0.02	0.01			
	M2	1.45	0.98	0.99	0.02	0.02	M2–M1	0.00	0.00
	M3	1.22	0.98	0.97	0.03	0.04	M3–M2	0.00	0.01
OSC	M1	1.42	0.98	0.99	0.02	0.02			
	M2	1.05	0.98	0.98	0.02	0.02	M2–M1	0.00	0.00
	M3	0.74	0.99	0.98	0.03	0.04	M3–M2	0.00	0.00

OI, occupational identity, OSC, occupational stigma consciousness, M1, configural invariance, M2, weak invariance, M3, strong invariance.

### 4.3 Stability and differences in occupational identity and occupational stigma consciousness among pre-service early childhood education teachers

A two-way repeated measures ANOVA with time, gender, and admission status as factors was conducted. Means and standard deviations were presented in Table 2. For occupational identity, the main effect of measurement time was significant (*F* = 24.71, *p* < 0.01, η^2^0 = 0.11). *Post-hoc* analyses revealed that occupational identity at T_1_ was significantly lower than that at T_2_ (*p* < 0.01), T_3_ (*p* < 0.01), and T_4_ (*p* < 0.01). Additionally, occupational identity at T_2_ was significantly lower than it was at T_3_ (*p* < 0.01). The main effect of gender was also significant (*F* = 7.10, *p* < 0.05, η^2^0 = 0.15), with male pre-service early childhood education teachers reporting significantly lower occupational identity than female pre-service teachers at T_2_, T_3_, and T_4_. The time × gender interaction was not significant (*F* = 0.65, *p* > 0.05, η^2^0 = 0.02). Regarding admission status, its main effect was significant (*F* = 11.92, *p* < 0.01, η^2^0 = 0.27). Across all four waves, pre-service early childhood education teachers with non-reassigned admission consistently demonstrated higher occupational identity than those with reassigned admission. The time × admission status interaction was not significant (*F* = 1.76, *p* > 0.05, η^2^0 = 0.06).

**Table 2:** Means and standard deviations of occupational identity and occupational stigma consciousness.

**Variable**	**Time**	**Male**	**Female**	**Reassigned admission**	**Non-reassigned admission**	**Total**
Occupational identity	T_1_	2.88 ± 0.45	3.36 ± 0.78	2.55 ± 0.45	3.46 ± 0.69	3.25 ± 0.74
	T_2_	3.04 ± 0.37	3.77 ± 0.56	2.97 ± 0.31	3.33 ± 0.47	3.60 ± 0.62
	T_3_	3.42 ± 0.26	3.98 ± 0.61	3.41 ± 0.29	3.63 ± 0.37	3.85 ± 0.60
	T_4_	3.28 ± 0.42	3.89 ± 0.67	3.28 ± 0.35	3.53 ± 0.57	3.75 ± 0.67
Occupational stigma consciousness	T_1_	3.92 ± 0.28	3.43 ± 0.63	3.92 ± 0.22	3.43 ± 0.64	3.54 ± 0.60
	T_2_	3.88 ± 0.25	3.18 ± 0.57	3.94 ± 0.18	3.16 ± 0.55	3.34 ± 0.59
	T_3_	3.54 ± 0.19	3.04 ± 0.51	3.54 ± 0.25	3.04 ± 0.50	3.16 ± 0.50
	T_4_	3.79 ± 0.34	3.19 ± 0.48	3.75 ± 0.33	3.20 ± 0.50	3.33 ± 0.52

T_1_, T_2_, T_3_, and T_4_ represent the four measurement time points.

For occupational stigma consciousness, the main effect of measurement time was significant (*F* = 11.94, *p* < 0.01, η^2^0 = 0.06). *Post-hoc* comparisons indicated that occupational stigma consciousness at T_1_ was significantly higher than that at T_2_ (*p* < 0.01), T_3_ (*p* < 0.05), and T_4_ (*p* < 0.01). The occupational stigma consciousness at T_2_ was also significantly higher than it was at T_3_ (*p* < 0.01), and occupational stigma consciousness at T_4_ was higher than it was at T_3_ (*p* < 0.01). The main effect of gender was significant (*F* = 10.13, *p* < 0.01, η^2^0 = 0.24), with male pre-service early childhood education teachers reporting higher occupational stigma consciousness than female pre-service teachers across all four waves. The time × gender interaction was not significant (*F* = 0.81, *p* > 0.05, η^2^0 = 0.02). Regarding admission status, the main effect of admission status was significant (*F* = 10.45, *p* < 0.01, η^2^0 = 0.25). Across all four waves, pre-service early childhood education teachers with reassigned admission consistently demonstrated higher occupational stigma consciousness than those with non-reassigned admission. The time × admission status interaction was not significant (*F* = 1.57, *p* > 0.05, η^2^0 = 0.05).

### 4.4 Correlational analysis between occupational stigma consciousness and occupational identity

The correlation coefficients among the four waves of occupational identity for pre-service early childhood education teachers ranged from 0.72 to 0.86, whereas those among the four waves of occupational stigma consciousness ranged from 0.56 to 0.93. The concurrent correlations between occupational identity and occupational stigma consciousness ranged from −0.88 to −0.75, and the cross-temporal correlations ranged from −0.85 to −0.72 (see Table 3).

**Table 3:** Correlation matrix for occupational identity and occupational stigma consciousness across Four waves.

**Variable**	**1**	**2**	**3**	**4**	**5**	**6**	**7**	**8**
1. OI T_1_	–							
2. OI T_2_	0.84^**^	–						
3. OI T_3_	0.77^**^	0.86^**^	–					
4. OI T_4_	0.72^**^	0.72^**^	0.86^**^	–				
5. OSCT_1_	−0.75^**^	−0.84^**^	−0.72^**^	−0.61^**^	–			
6. OSCT_2_	−0.79^**^	−0.88^**^	−0.85^**^	−0.77^**^	0.82^**^	–		
7. OSC T_3_	−0.85^**^	−0.78^**^	−0.88^**^	−0.87^**^	0.71^**^	0.86^**^	–	
8. OSC T_4_	−0.77^**^	−0.72^**^	−0.81^**^	−0.87^**^	0.56^**^	0.77^**^	0.93^**^	–

OI, occupational identity, OSC, occupational stigma consciousness, T_1_, T_2_, T_3_, and T_4_ represent the four measurement time points, ^**^p < 0.01.

### 4.5 Cross-lagged analysis between occupational stigma consciousness and occupational identity

The results indicated that, after controlling for T_1_ occupational identity, T_1_ occupational stigma consciousness significantly predicted T_2_ occupational identity (β = −0.47, *p* < 0.01). Conversely, after controlling for T_1_ occupational stigma consciousness, T_1_ occupational identity significantly negatively predicted T_2_ occupational stigma consciousness (β = −0.40, *p* < 0.01).

After controlling for T_2_ occupational identity, T_2_ occupational stigma consciousness significantly negatively predicted T_3_ occupational identity (β = −0.43, *p* < 0.01). However, after controlling for T_2_ occupational stigma consciousness, T_2_ occupational identity did not significantly predict T_3_ occupational stigma consciousness (β = −0.07, *p* > 0.05).

**Figure 1:**
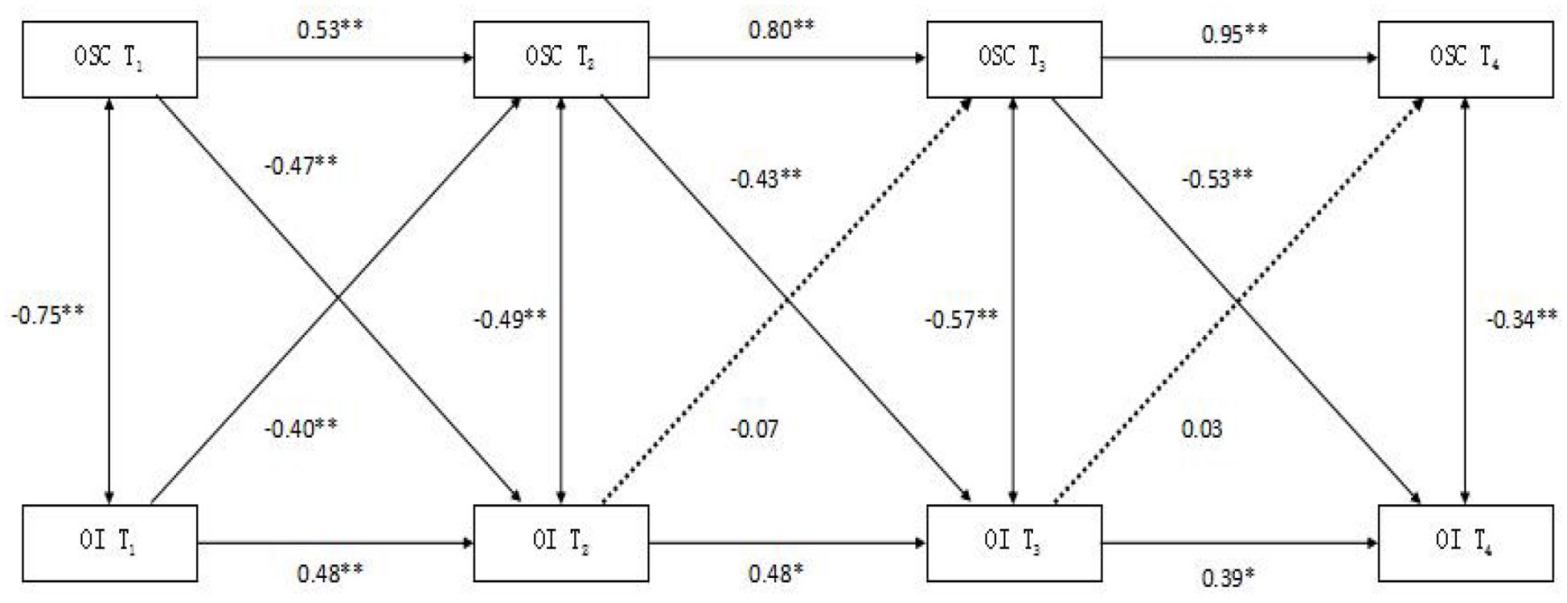
Cross-lagged outcome analysis of occupational identity and occupational stigma consciousness. OI, occupational identity; OSC, occupational stigma consciousness, T_1_, T_2_, T_3_, and T_4_ represent the four measurement time points, **p < 0.0.

Furthermore, after controlling for T_3_ occupational identity, T_3_ occupational stigma consciousness significantly predicted T_4_ occupational identity (β = −0.53, *p* < 0.01). In contrast, after controlling for T_3_ occupational stigma consciousness, T_3_ occupational identity did not significantly predict T_4_ occupational stigma consciousness (β = 0.03, *p* > 0.05; see Figure 1).

## 5 Discussion

### 5.1 The stability of occupational identity and occupational stigma consciousness

We found that the development of occupational identity among pre-service early childhood education teachers follows an initial increase, then stabilizes. Occupational identity was lowest during the freshman year. It remained significantly lower in the sophomore year than in the junior year, whereas no significant difference was observed between the junior and senior years. These developmental patterns are consistent with stage theories of occupational socialization, which posit that occupational formation occurs in phases. On this basis, Hypothesis 1 was rejected. Our data further reveal a notable transition in the senior year. Prior cross-sectional studies have indicated that students' occupational identity tends to improve significantly during the early stages of their academic program (Flores, 2020; Hong et al., 2017; Tomlinson and Jackson, 2021). At the same time, other research suggests that internships can shift pre-service early childhood education teachers' perceptions from idealism toward realism, and that setbacks during practice can lead to a decline in occupational identity by the senior year (Hong et al., 2017; Kelchtermans and Ballet, 2002). In recent years, however, China has implemented policy measures to strengthen guidance for teaching practicum (Cai et al., 2022). These interventions likely enhance the quality of practicum experiences and mitigate common training-related challenges. As a result, even after completing their practicum, senior-year pre-service teachers in our sample showed no significant drop in occupational identity. Instead, identity stabilized in the final year, suggesting a phase of narrative consolidation. Overall, these findings align with the stage theory of occupational socialization (Schein, 1978). Occupational identity develops sequentially: the early rise reflects integration of skills and knowledge, while the subsequent plateau corresponds to narrative stabilization.

This study found that occupational stigma consciousness among pre-service early childhood education teachers initially declines, then subsequently increases. Stigma consciousness peaked during the freshman year and reached its lowest point in the junior year. Distinct cognitive and emotional mechanisms appear to operate in the perception of stigma compared to identity development. As a result, Hypothesis 1 was rejected. Previous cross-sectional research indicates that early childhood education teachers perceive a high level of social stigma associated with their occupation (Bo et al., 2022). The peak in stigma consciousness during the freshman year reflects an acute phase of stigma salience, during which public narratives strongly shape early perceptions. In recent years, media coverage of child abuse cases involving early childhood education teachers has risen annually. For example, in 2017, over 40,000 news articles on such incidents were published on Baidu News, a major Chinese online news platform. By 2019, this number had increased to more than 80,000 (Chen and Xiong, 2019). Participants in this study were enrolled in 2020. The extensive media attention to early childhood teachers' deviant conduct before their enrollment likely heightened their initial occupational stigma consciousness. Furthermore, China has intensified its crackdown on child abuse in recent years, leading to a significant decline in severe child maltreatment cases (Zhang L. et al., 2020). Additionally, through deeper theoretical engagement, pre-service early childhood education teachers were able to rationally analyze negative occupational information using acquired knowledge, which likely contributed to a significant decline in occupational stigma consciousness during the sophomore and junior years. As they approach employment, senior-year students engage in more profound reflection on the status of their occupation (Dome et al., 2005; Kearney, 2014). This can be understood as an anticipatory reality shock (Veenman, 1984). Confronting the job market makes the occupation's structural devaluation more tangible (Ashforth and Kreiner, 1999), reactivating stigma consciousness and leading to the observed rebound in the senior year. This rebound aligns with stage theories of occupational socialization, which posit that occupational development unfolds in distinct phases (Schein, 1978). The senior year marks a critical transition during which students prepare for entry into the workforce. This transition increases their vulnerability to occupational realities. Reality shock theory offers further explanation (Veenman, 1984). Idealized training environments give way to workplace realities. During this phase, students directly encounter the job market and societal perceptions, making the structural devaluation of the occupation more salient. As a result, occupational stigma consciousness reemerges.

### 5.2 Gender and admission status differences in occupational identity and occupational stigma consciousness

Cross-sectional studies indicate that male pre-service teachers exhibit lower occupational identity than female teachers (Kremer-Hayon et al., 2002; Moore and Hofman, 1988). In this study, we found that over the 4 years, male pre-service early childhood education teachers consistently reported higher levels of occupational stigma consciousness than their female peers. Hypothesis 2 was therefore supported. This gender disparity parallels findings from other non-Western contexts. For example, phenomenological research with male preschool teachers in Turkey reveals similar experiences of navigating societal prejudice and justifying their career choice within traditional gender norms (Akyol et al., 2023). Such cross-societal consistency suggests that the intersection of gender and occupational stigma represents a widespread structural barrier across cultural settings. The early childhood teaching occupation is often viewed as misaligned with traditional expectations for men's careers (Kyriacou and Kunc, 2007; Richardson and Watt, 2006). These social expectations define normative gender roles (Holland, 1963). For male students, this misalignment creates a dual burden: they face stigma both for their chosen occupation and for deviating from gender norms. This dual awareness can be understood as a form of double consciousness regarding occupational stigma (Pinel, 1999). It heightens sensitivity to negative stereotypes and fosters chronic role conflict. Together, these processes meaningfully hinder the formation of a stable occupational identity.

This study found that over the 4 years, pre-service early childhood education teachers with reassigned admissions exhibited lower occupational identity and higher occupational stigma consciousness than those with non-reassigned admissions. Hypothesis 2 was supported. Compared to their non-reassigned peers, reassigned students often enter the career as a non-first-choice. Studies confirm that they show less initial interest and may hold certain prejudices (Biggs and Telfer, 1993; Jing et al., 2015). This initial disposition can be understood through expectancy-value theory (Borders et al., 2004). They begin with lower attainment value (seeing less worth in the career) and higher perceived costs (anticipating greater social or personal sacrifices). Consequently, their initial occupational commitment is weaker, and their skepticism is higher. This initial setup creates a path of disadvantage. Low commitment hinders the deep engagement needed for identity building. Preexisting skepticism acts as a filter, making societal stigma more salient and credible, as predicted by stigma consciousness theory (Pinel, 1999). A self-reinforcing cycle emerges. Initial conditions in occupational preparation create pathways that shape long-term developmental trajectories. This persistent gap can be understood through expectancy-value theory and the concept of path dependency (Borders et al., 2004). Students with reassigned admission often enter with lower initial commitment. This sets a lower baseline for occupational identity development. Concurrently, they hold higher sensitivity to social devaluation. This amplifies their occupational stigma consciousness. Over time, these initial conditions create a self-reinforcing cycle. Early disadvantages in engagement and perception solidify. They form stable developmental trajectories throughout the 4-year program.

### 5.3 Cross-lagged associations between occupational identity and occupational stigma consciousness

Our longitudinal analysis demonstrated that occupational stigma consciousness consistently and negatively predicted subsequent occupational identity across all 4 years. This supports Hypothesis 3. Our finding extends previous cross-sectional evidence on the detrimental role of stigma (Lai et al., 2013; Roitenberg, 2020; Schaubroeck et al., 2018). It also aligns with Folkman and Lazarus (1985) transactional model of stress (Folkman and Lazarus's, 1985). Within this framework, stigma consciousness functions as a recurrent stressor. It triggers threat appraisals. These appraisals then activate coping responses. Often, these responses are maladaptive. They gradually erode occupational identity. This erosion has practical significance, as diminished occupational identity is linked to more negative teaching emotions and a higher risk of burnout (Hai et al., 2023; Li et al., 2025). The cross-lagged pattern showed notable asymmetry. Occupational stigma consciousness persistently predicted identity. In contrast, identity's influence on stigma was time-limited. Identity significantly reduced stigma consciousness from the freshman to the sophomore year. However, this effect did not continue in subsequent years. This pattern leads us to reject Hypothesis 4. Savickas's (2019) career construction theory helps explain both the early protective effect and its subsequent diminishment (Savickas, 2019). A strong initial occupational identity likely supplies narrative and psychological resources. These resources enable students to reframe or resist stigmatizing perceptions. This occurs during the formative adaptability phase of occupational development. Yet this capacity for narrative reconstruction appears to diminish in later years. Cognitive dissonance theory clarifies this pattern (Festinger, 1957). Freshmen hold both early commitment and stigma awareness. This creates psychological tension. To reduce dissonance, they may devalue the stigma. This explains the T_1_ → T_2_ predictive path. However, repeated exposure leads to stigma internalization (Link and Phelan, 2001). Stigma becomes embedded in the self-concept. Individual reframing loses potency. Thus, identity no longer predicts stigma in later years (T_2_ → T_3_, T_3_ → T_4_).

The occupational context of early childhood education further clarifies these dynamics. This occupation has relatively low societal prestige and faces structural disadvantages in authority, income, and requirements (Zhang L. et al., 2020). According to occupational stigma theory, such roles are systemically devalued (Ashforth and Kreiner, 1999). This systemic devaluation creates a fundamental lack of the social and material resources that typically buffer against stigma (Sharma et al., 2022). Consequently, practitioners must rely more heavily on individual psychological resources. However, cognitive dissonance theory reveals the instability of this reliance (Festinger, 1957). Occupational identity encompasses both intrinsic value (e.g., meaning) and extrinsic value (e.g., status; Kielhofner, 1985). Stigma directly attacks both dimensions. To maintain a positive identity amidst this, individuals experience psychological tension (dissonance). Initially, they may resolve this by dismissing the stigma's validity—a viable strategy when identity is malleable and occupational immersion is high (Gurr, 1970; Kreiner et al., 2006). Yet, as training progresses, the reality of structural constraints becomes inescapable (Zhang X. A. et al., 2020). The low prestige and pay continually reinforce the stigma. Here, the dissonance resolution strategy flips. Sustaining a positive self-view in the face of overwhelming structural evidence becomes increasingly aversive. The psychologically less taxing path is to accommodate the stigma as a fixed occupational reality, thereby decoupling self-worth from external devaluation. This process reflects the core premise of occupational stigma theory: individual coping tactics exist, but systemic devaluation persists and ultimately dominates (Ashforth and Kreiner, 1999). Even proactive efforts yield limited results (Holland, 1963). Thus, the initial protective effect of identity, fueled by dissonance reduction, diminishes. Pre-service teachers tacitly recognize the limits of individual agency in the face of systemic forces. This explains why identity loses its predictive power for stigma consciousness after the 1st year, accounting for the non-significant relationships observed from sophomore through senior years. This diminishing protective effect may be further understood through a cultural lens. The career construction theory (Savickas, 2019), developed in Western individualistic contexts, emphasizes personal narrative reconstruction as a key resource for adaptability. However, in collectivistic societies like China, individuals may be more inclined to perceive occupational stigma as a fixed social reality to be managed or endured, rather than a narrative to be actively rewritten through individual agency (Markus and Kitayama, 2014). This cultural tendency, also observed in the comparative study of preschools in Japan and China, might explain why the initial, internally-driven identity buffer loses its potency against persistent external stigma over time (Tobin et al., 2009). Thus, early identity can offer initial protection. Yet the persistent, structurally embedded nature of occupational stigma ultimately exerts a stronger influence. This influence shapes the occupational self-concept of pre-service early childhood teachers throughout their training.

### 5.4 Limitations and future directions

This study has several limitations. First, the sample was drawn from a single institution, which may limit the generalizability of the findings. Although the institution is representative of regional teacher education universities in China, and the program's enrollment size (e.g., 52 students in the 2020 cohort) is typical of comparable programs, the final longitudinal sample of 34 participants restricts statistical power and the ability to conduct more nuanced subgroup analyses. For example, findings regarding gender differences rely on only 8 male participants and must be interpreted with caution. While this mirrors the pronounced gender imbalance in China's early childhood education workforce (e.g., a female-to-male ratio of 44:1 among in-service teachers), future research necessitates larger, multi-institutional samples to validate these preliminary trends and enable robust analysis of underrepresented groups.

Second, our analytical approach warrants discussion. The cross-lagged panel model was chosen to explicitly test reciprocal predictions over time, which was central to our research question. It provides a robust estimate of longitudinal associations at the level of observed variables. However, this model does not statistically separate between-person stability from within-person change. Therefore, the observed cross-lagged effects may partially reflect time-invariant individual differences. Our results should be interpreted as demonstrating temporal precedence and reciprocal prediction, rather than definitive within-person causality. Future studies could advance this line of inquiry by employing models that disaggregate these variance components, such as the Random Intercept Cross-Lagged Panel Model.

Third, the measurement of occupational stigma consciousness merits note. The scale we employed has been validated with in-service early childhood teachers in China. This provided a basis for its use in our study. However, its psychometric properties for the pre-service population require further confirmation. Developing more context-sensitive measures would strengthen future research.

Additionally, the reliance on self-report measures may introduce common method variance, though Harman's single-factor test indicated this was not a substantial concern in the current data. Future studies could incorporate mixed-methods approaches, such as interviews or observational data, to enrich understanding of how stigma and identity evolve in context.

Finally, this study was conducted within a specific cultural and policy setting—China's centralized teacher education system. Cross-cultural comparisons are needed, especially with other non-Western contexts. These regions often face similar status challenges in early childhood education. Such comparisons can clarify whether the findings are unique to this context or reflect broader trends in teacher development. Such comparisons would help distinguish context-specific findings from broader patterns in pre-service teacher development.

## 6 Conclusions

The occupational identity of pre-service early childhood education teachers followed an initial upward trend, then stabilized. In contrast, their occupational stigma consciousness initially declined, then increased. Over the 4 years, significant differences were observed in both occupational identity and occupational stigma consciousness across gender and admission status. Furthermore, baseline occupational stigma consciousness consistently and negatively predicted later occupational identity across all waves. Specifically, occupational identity at T_1_ significantly and negatively predicted occupational stigma consciousness at T_2_, whereas occupational identity at T_2_ and T_3_ did not predict occupational stigma consciousness at T_3_ and T_4_, respectively.

Teacher education programs should implement targeted interventions, particularly for male pre-service early childhood education teachers and those with reassigned admissions, to strengthen occupational identity and mitigate occupational stigma-related effects. These interventions should be structured developmentally. In the freshman and sophomore years, enhanced occupational value education should integrate stigma-deconstruction modules into foundational courses, while improved practical training quality should include early, structured observation sessions across diverse preschool settings. In junior and senior years, psychological support should evolve into practicum-linked supervision groups focusing on real-time coping strategies against societal stereotypes. For male pre-service early childhood education teachers, mentoring programs with experienced male early childhood education teachers are recommended. For pre-service early childhood education teachers with reassigned admissions, early-semester occupational identity workshops and affinity group discussions can address initial career ambivalence.

## Data Availability

The original contributions presented in the study are included in the article/supplementary material, further inquiries can be directed to the corresponding author.
